# Comprehensive assessment of activity, specificity, and safety of hypercompact TnpB systems for gene editing

**DOI:** 10.1186/s13059-026-03949-8

**Published:** 2026-01-21

**Authors:** Changchang Xin, Guanghai Xiang, Shiwei Cao, Yuhong Wang, Shaopeng Yuan, Xinyi Liu, Yongyuan Huo, Jing Sun, Xichen Wan, Duan Liu, Jiaxu Hong, Jiazhi Hu, Haoyi Wang

**Affiliations:** 1 State Key Laboratory of Gene Function and Modulation Research, School of Life Sciences, PKU-THU Center for Life Sciences, Peking University, Beijing, 100871 China; 2 State Key Laboratory of Organ Regeneration and Reconstruction, Institute of Zoology, Chinese Academy of Sciences, Beijing, 100101 China; 3 Institute of Health and Medicine, Hefei Comprehensive National Science Center, Hefei, Anhui 230601 China; 4 Beijing Institute for Stem Cell and Regenerative Medicine, Beijing, 100101 China; 5 University of Chinese Academy of Sciences, Beijing, 100049 China; 6 Peking University Chengdu Academy for Advanced Interdisciplinary Biotechnologies, Chengdu, Sichuan 610213 China; 7 Department of Ophthalmology, Eye & ENT Hospital, State Key Laboratory of Brain Function and Disorders, MOE Frontiers Center for Brain Science, Fudan University, Shanghai, 200031 China; 8 Shanghai Key Laboratory of Rare Disease Gene Editing and Cell Therapy, Shanghai, 200032 China; 9 Shanghai Engineering Research Center of Synthetic Immunology, Shanghai, 200032 China

## Abstract

**Background:**

As the ancestor of CRISPR-Cas12 nucleases, TnpB represents the most compact gene editing tool currently available. Recent studies have identified multiple TnpB systems with gene editing activity in mammalian cells, and the potential of TnpB in treating diseases has been demonstrated in animal models. However, the editing characteristics of various TnpB systems, comparable to CRISPR tools, require more extensive investigation.

**Results:**

Using a standardized evaluation framework, we conduct a thorough analysis of the editing properties of four TnpB variants alongside representative Cas12 and Cas9 tools applications. Overall, TnpBs exhibit intermediate editing activity and safety profiles among all tested systems, with *IS*Ymu1 TnpB demonstrating a good performance in both editing activity and specificity. Considering its compact size, potent editing efficiency and high specificity, *IS*Ymu1 TnpB represents a promising candidate for gene therapy.

**Conclusions:**

By comprehensively analyzing genome editing outcomes, we characterize TnpB systems for genome editing and identify *IS*Ymu1 TnpB as an optimal miniature RNA-guided genome editors with balanced performance, highlighting its potential for therapeutic applications.

**Supplementary Information:**

The online version contains supplementary material available at 10.1186/s13059-026-03949-8.

## Background

CRISPR technology holds great potential in clinical translation [1–6], exemplified by the approval of the world's first CRISPR gene editing therapy (Casgevy) by the MHRA and FDA at the end of 2023 [7, 8]. Adeno-associated virus (AAV) has become a prevalent in vivo delivery approach due to its clinical validation and its ability to target a variety of clinically relevant tissues [9]. However, classical Cas9 or Cas12 proteins, which are over 1,200 amino acids (aa) in size, are generally too large to fit into a single AAV, which has a cargo size limit of approximately 4.7 kilobases (kb) [10]. This limitation is especially challenging for base editing (BE) and prime editing (PE) systems, where additional fusion modules further complicate delivery [11–14]. Recent development of small Cas9 and Cas12 homologs such as *Sa*Cas9 [15], *Cj*Cas9 [16], and e*Nme2*-Cas9 [17], Cas12e (CasX) [18, 19], Cas12f [20–24], Cas12j (CasΦ) [25, 26], Cas12l (Casπ) [27], and Cas12n [28], have provided potential solutions. Additionally, the ancestors of CRISPR-Cas9 and Cas12 nucleases, IscB and TnpB, associated with the prokaryotic IS200/IS605 transposon family, have demonstrated gene editing capabilities, representing the most compact RNA-guided gene editing tools to date [29–32].

*IS*Dra2 (408aa) form *Deinococcus radiodurans* is the first identified TnpB with gene editing activity in human cells [29]. Several studies have elucidated its structure [30, 31], optimized TnpB protein and reRNA [33, 34], and applied it to treat tyrosinaemia mouse model [33]. Recently we established a pipeline for de novo annotation, prediction, and experimental identification of active TnpB systems from prokaryotic genomes, and identified five TnpB systems with editing activity in human cells [35]. However, a systematic characterization of the editing characteristics and specificity of these TnpB gene editors has not been conducted, and a rigorous comparison of the performance of TnpB systems with well-established CRISPR tools is still lacking.

Typically, gene editors induce a targeted double-strand break (DSB) in the genome, leveraging endogenous DNA DSB repair mechanisms to achieve editing outcomes. Primer-extension-mediated sequencing (PEM-seq) utilizes a bait-primer designed on one side of the broken ends to capture prey ends, enabling the assessment of insertions/deletions (indels) at the target sites, as well as the harmful chromosomal rearrangements like large deletions, exogenous DNA fragment insertions, and chromosomal translocations between the bait broken ends and other prey ends [36–38].

In this study, we comprehensively analyzed four TnpB systems (including the original *IS*Dra2 and three newly identified TnpBs: *IS*Dge10, *IS*Aam1, *IS*Ymu1) and representative members from the Cas12 and Cas9 families (including *As*Cas12a, CasMINI, *Sp*Cas9, and the miniature Cas9 homolog e*Nme2*-C.NR) using PEM-seq, to evaluate their editing activity, specificity, and safety.

## Results

### Assessment of the editing outcomes of TnpB systems by PEM-seq

**Fig. 1:**
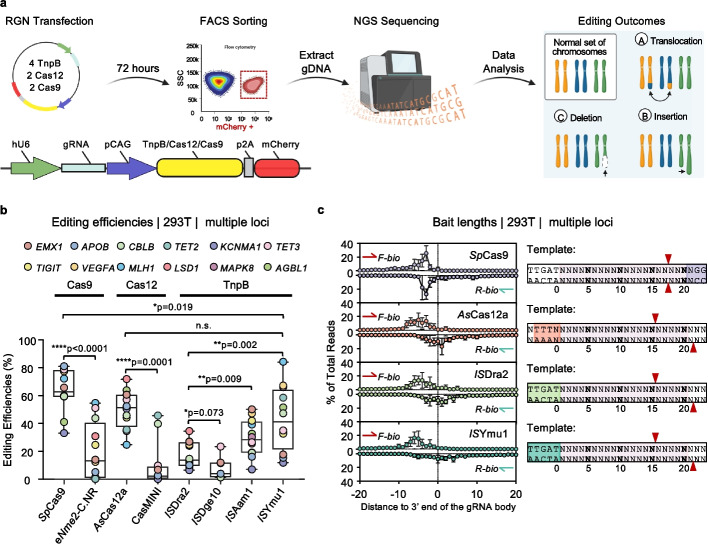
Evaluation of TnpB and Cas9, Cas12 by PEM-seq at multiple loci. **a** Schematics showing the assessment of TnpB, Cas9, and Cas12 by PEM-seq. HEK293T cells were transfected with plasmids containing both single guide (sg) RNA and TnpB, Cas12, or Cas9 nuclease coexpressed with mCherry fluorescent protein, and successfully transfected cells were sorted by FACS at 72 h post-transfection, followed by PEM-seq library construction. PEM-seq could simultaneously identify translocations, deletions, and insertions. **b** Summary dot plots showing the editing efficiencies of the eight representative TnpB/Cas12/Cas9 nucleases at the indicated twelve loci in HEK293T cells as detected by PEM-seq. Editing efficiency is calculated as the total percentage of insertions, deletions, and translocations. Whiskers indicate values ranging from the minimum to the maximum, while the box borders indicate the first and third quartiles (*N* = 12). Data are presented as mean ± SD, the median is indicated by a vertical line through the box.Source data can be found in the Source Data file. Paired two-tailed t-test, n.s., not significant, *p ≤ 0.05, **p ≤ 0.01, ***p ≤ 0.001, ****p ≤ 0.0001. **c** Left: the location distribution and frequency of bait broken ends across all effective editing sites are depicted. The bait broken ends are categorized as target-strand broken ends and non-target-strand broken ends based on the design direction of the Bio-primer. The horizontal axis indicates specific positions, with black dotted lines marking the 3’ end of the gRNA body. The vertical axis represents the probability of bait broken ends mapping at that exact location. Right: schematic diagrams illustrating speculated double-stranded DNA cleavage patterns induced by specific nucleases as inferred from the distribution of bait broken ends. The major minor cleavage sites are indicated by red arrowheads, while the PAM sequences of various nucleases are highlighted with different colors

To systematically assess and compare the editing characteristics of TnpB, Cas9, and Cas12 systems, we selected four TnpB tools, including the first established *IS*Dra2-TnpB and three newly identified TnpBs (*IS*Dge10, *IS*Aam1, *IS*Ymu1) [35], along with two Cas12 (*As*Cas12a and the engineered Cas12f variant CasMINI-V3.1 with optimized sgRNA design 2 (hereafter CasMINI) [24]) and two Cas9 tools (the commonly used *Sp*Cas9 and the compact Cas9 variant e*Nme2*-C.NR). We cloned these eight tools into the same backbone vector, linking a mCherry reporter gene to the enzyme through a P2A self-cleavage peptide (Fig. 1a). To ensure comparability despite their different target adjacent motif (TAM, also known as protospacer adjacent motif, PAM) requirements, we carefully selected 12 genomic loci for editing, except for 7 sites for *IS*Dge10 due to the restriction of “TTAT” TAM within the other five sites. For each system, guide RNA (gRNA) was designed to target sequences overlapping within a narrow range (< 52 bp) (Additional file 1: Fig. S1).

The plasmid encoding each nuclease and its corresponding gRNA was transfected into HEK293T cells. After 72 h, the mCherry-positive cells were collected by FACS for genomic DNA extraction. The editing outcomes were characterized by PEM-seq method [36, 37] (Fig. 1a). We first evaluated the editing efficiency of all tested systems by quantifying the ratio of editing products and byproducts (including deletions, insertions, and translocations) to the total number of sequencing target alleles. While the editing efficiencies of *IS*Dra2, *IS*Aam1, and *IS*Ymu1 TnpB systems (13.7%, 28.3%, and 41.1%, respectively) were lower than those of the widely used *Sp*Cas9 (62.8%) and *As*Cas12a (51.3%), they displayed effective cleavage at all 12 tested sites. In contrast, *IS*Dge10 effectively edited only 3 out of the 7 tested sites, with a median efficiency of 4.0%. Consistent with previous observations, the *IS*Aam1 and *IS*Ymu1 TnpB systems exhibited higher editing efficiencies than the compact Cas9 variant e*Nme2*-C.NR (median of 13.2%) and the miniature Cas12f family nuclease CasMINI, which effectively edited only 4 of the 12 tested sites with a median efficiency of 2.2% (Fig. 1b) [35].

To allow a more rigorous comparison between TnpB systems with *Sp*Cas9, we analyzed target sites that carried identical protospacer sequences shared between selected nuclease pairs. For the comparison between *Sp*Cas9 and *IS*Aam1, we designed 8 gRNAs targeting 8 endogenous loci, following the sequence pattern of TTTAA(N20)NGG. Regarding the comparison among *Sp*Cas9, *IS*Dra2, and *IS*Ymu1, we designed another set of 8 gRNAs with the pattern TTTAT(N20)NGG. Following plasmid transfection, genomic DNA was extracted and analyzed by NGS. In the head to head comparison of *Sp*Cas9 vs. *IS*Aam1, *Sp*Cas9 exhibited significantly higher activity (median 48.5%) than *IS*Aam1 (median 19.9%), with *IS*Aam1 achieving successful editing at 5 out of the 8 tested sites (Additional file 1: Fig. S2a). In the *Sp*Cas9 vs. *IS*Dra2/*IS*Ymu1 comparison, *Sp*Cas9 again outperformed the TnpB systems (median 34.6% versus 11.6% for *IS*Dra2 and 18.0% for *IS*Ymu1). Notably, *IS*Ymu1 consistently demonstrated higher activity than *IS*Dra2 across the majority of targets (Additional file 1: Fig. S2b). These findings confirm that *Sp*Cas9 achieves the highest editing efficiency among the systems tested, while *IS*Ymu1demonstrates moderately higher efficiency than *IS*Dra2.

Next, we characterized the cleavage pattern of each system. By aligning the position of the bait broken ends at all effective cleavage sites, we found that *IS*Dra2 and *IS*Ymu1 predominantly cleave DNA approximately 15 nt downstream of the TAM on the non-target strand (NTS) and 1 nt downstream of the spacer on the target strand (TS), resulting in an approximate 6-nt sticky end (Fig. 1c). Despite recognizing a different TAM (5’-TTTAA versus 5’-TTTAT for *IS*Dra2 and *IS*Ymu1), *IS*Aam1 exhibited a similar cleavage pattern, with predominant cleavage positions on the NTS lay at approximately 16 nt downstream of the TAM (Additional file 1: Fig. S2c). This cleavage pattern closely resembles the staggered cleavage of the Cas12 family [31]. Consistent with previous studies [17, 39], the distribution of bait broken ends for *Sp*Cas9 and e*Nme2*-C.NR reflects their cleavage at 3 nt upstream of the PAM, resulting in a blunt end (Fig. 1c and Additional file 1: Fig. S2c).

Altogether, the above characterization implies that TnpB systems share similarities in cleavage mechanisms with the Cas12 family, and *IS*Dra2, *IS*Aam1, and *IS*Ymu1 TnpBs exhibit robust editing activity across all tested target sites.

### Characterization of deletion events

**Fig. 2:**
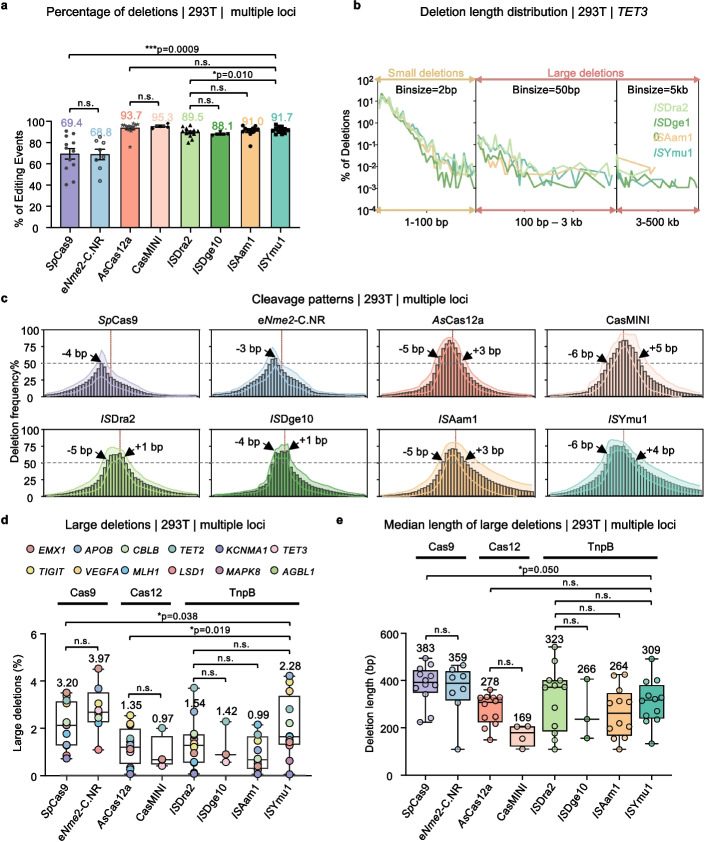
Similar to the Cas12 family, TnpB nucleases generate more deletional products than Cas9. **a** Percentage of deletions for the indicated nucleases as detected by PEM-seq. The numbers indicate the average percentage of deletion fragments to total editing events across all effective editing sites where the editing efficiency exceeds 5% (Data are presented as mean ± SD). Paired two-tailed t-test, n.s., not significant, *p ≤ 0.05, ***p ≤ 0.001. **b** Distribution pattern of deletion junctions at the *TET3* locus in HEK293T cells. Total deletions were categorized into small (< = 100 bp, yelow) and large deletions (> 100 bp, red), and further subdivided into 3 regions: junctions within 100 bp from the cut site; junctions ranging from 100 bp to 3 kb downstream from the cut site; and junctions ranging from 3 to 500 kb downstream from the cut site. Please note that bin sizes of 2 bp, 50 bp, and 5 kb were applied, respectively. **c** The location distribution and frequency of nucleotide deletion events surrounding the target sequence are depicted. The horizontal axis indicates specific positions, with red dotted lines marking the 3’ end of the gRNA body. The vertical axis represents the probability of nucleotide deletion events occurring at that exact location, with black dotted lines marking the 50% boundary lines. The positions susceptible to nucleotide deletion events across all tested loci are indicated by the black arrow, with their positions relative to the 3’ end of the gRNA body marked. **d**, **e** The percentage of large deletions relative to total editing events (**d**) and the median length distribution of large deletions (**e**) for these nucleases in HEK293T cells, as detected by PEM-seq. Box plots show quartiles with whiskers (min to max). (Data are presented as mean ± SD). The numbers above the bars indicate the average median length of large deletions (**e**). Paired two-tailed t-test, n.s., not significant, *p ≤ 0.05

As deletions are usually the main editing products, we next characterized deletions generated by TnpB systems versus other editing tools at tested sites. The four TnpB systems predominantly caused nucleotide deletions, ranging from 88.1% to 91.7%. These rates were slightly lower than those induced by Cas12 systems (93.7% for *As*Cas12a; 95.3% for CasMINI), but significantly higher than those induced by the Cas9 system (69.4% for *Sp*Cas9; 68.8% for e*Nme2*-C.NR) (Fig. 2a).

Further analysis of deletion length distributions reveals that the majority of products generated by the four TnpB systems are small deletions (< 100 bp), primarily concentrated between 0–40 bp (Fig. 2b and Additional file 1: Fig. S3a-c). Zoomed-in view of nucleotide deletions at positions surrounding the target sequence reveals distinct patterns: for the Cas9 family, which symmetrically cleaves DNA on both strands, the probability of nucleotide deletions at each position gradually decreases with increasing distance from the cleavage site. In contrast, for the Cas12 and TnpB families, which cleave DNA at alternate positions on the two strands, the probability of nucleotide deletions is higher between the cleavage sites on each strand. These distribution patterns of 0–40 bp deletions correlate well with the cleavage patterns (Fig. 2c and Additional file 1: Fig. S3c, d). With regards to 20–40 bp deletions, *IS*Ymu1 TnpB produces the highest proportion compared to the other 7 systems (Additional file 1: Fig. S3b).

Next, we characterized the large fragment deletion events (> 100bp). The percentage of large deletions relative to total editing events induced by *IS*Dra2, *IS*Dge10, *IS*Aam1, and *IS*Ymu1 TnpB systems were 1.54%, 1.42%, 0.99%, and 2.28%, respectively. These rates are lower than those observed with the Cas9 system (3.20% for *Sp*Cas9 and 3.97% for e*Nme2*-C.NR) but comparable to *As*Cas12a and CasMINI (1.35% and 0.97%, respectively) (Fig. 2d). Further analysis of the median lengths of large deletions across all eight systems indicates that *Sp*Cas9 has the highest median length at 383 bp, and CasMINI has the lowest at 169 bp, while the TnpB systems fall in the middle range (Fig. 2e). In summary, TnpB systems exhibit similar feature as the Cas12 family in generate deletions during editing.

### Characterization of insertion events

**Fig. 3:**
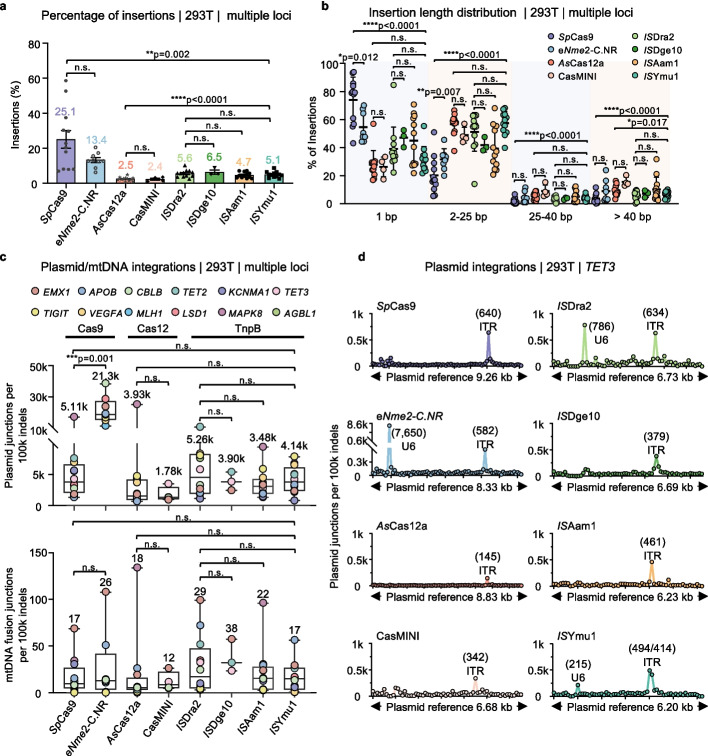
Plasmid cleavage and mitochondrial DNA integration of different nucleases. **a** The insertion percentages for the indicated nucleases as detected by PEM-seq. The numbers above the bars indicate the average percentage of insertion fragments across all effective editing sites where the editing efficiency exceeds 5% (Data are presented as mean ± SD). Paired two-tailed t-test, n.s., not significant, **p ≤ 0.01, ****p ≤ 0.0001. **b** The length distribution of insertions for all effective editing loci as detected by PEM-seq in HEK293T cells, where the vertical axis represents the percentage of insertions indicated relative to the total insertions, and the columns represent a subdivision into specific lengths: 1 bp, 2–25 bp, 25–40 bp, and > 40 bp. Paired two-tailed t-test, n.s., not significant, *p ≤ 0.05, **p ≤ 0.01, ****p ≤ 0.0001. **c** The junction numbers of plasmid integrations (top) and mtDNA integrations (bottom) per 100 k insertions and deletions (indels) for these nucleases at all effective editing loci in HEK293T cells, as detected by PEM-seq. K means thousands. Whiskers indicate values ranging from the minimum to the maximum, while the box borders indicate the first and third quartiles (Data are presented as mean ± SD). The median is indicated by a vertical line through the box, and the number above the box indicates the average junction numbers. Source data can be found in the Source Data file. Paired two-tailed t-test, n.s., not significant, ***p ≤ 0.001. **d** The mapping position distribution of vector integration junctions across the respective plasmids at the *TET3* locus as detected by PEM-seq. The vertical axis represents the junction numbers of plasmid integrations that occur exactly at the indicated position when down-sampled to 100 k indels. K means thousands. The bin size of the plasmid sequence is 100 bp. Junction numbers for the U6-sgRNA region and Adeno-associated virus (AAV) inverted repeat (ITR) regions are marked above separately

We next analyzed the nucleotide insertion events generated by these editing tools. Overall, Cas9 family members generated the highest percentage of insertional events, with 13.4% for e*Nme2*-C.NR and 25.1% for *Sp*Cas9, probably due to their predominance to induce 1-bp insertion (Fig. 3a, b). In contrast, TnpB systems generated insertions ranging from 4.7% to 6.5%, which is higher than those generated by the Cas12 family nucleases (2.4% to 2.5%). The distribution of insertion lengths induced by TnpB systems is similar to that of the Cas12 family, with the majority of insertions falling within 2–25 bp for the *IS*Dra2 and *IS*Ymu1 TnpB systems. Notably, all eight systems showed lower levels of insertions greater than 25 bp. Among them, CasMINI had the highest rate at 24.5%, while *Sp*Cas9 had the lowest at 5.6% (Fig. 3b and Additional file 1: Fig. S4a).

Previous studies suggest that large inserted DNA sequences may originate from delivery vectors or mitochondrial DNA (mtDNA) [40]. By aligning these inserted sequences with the corresponding nuclease-expressing vectors and mtDNA, we identified integrations of transfected plasmid DNA or mtDNA at nearly all the tested loci. The average rates of plasmid DNA integrations by TnpB across editing loci range from 3.48k to 5.26k per 100k indels, much higher than the levels of mtDNA integrations, ranging from 17 to 38 per 100k indels. The plasmid and mtDNA integration levels of TnpB enzymes are comparable to *Sp*Cas9 but slightly higher than those of the Cas12 family. Notably, e*Nme2*-C.NR exhibited extremely high levels of plasmid DNA integrations at all effective edited loci, averaging 21.3k plasmid insertions per 100k indels (Fig. 3c). Additionally, by mapping integrated plasmid fragments across their respective vectors, we observed a widespread distribution throughout the plasmid backbones across all eight systems, with accumulation at the fragile AAV inverted terminal repeat (ITR) region (ranging from 145 to 640 AAV ITR integrations per 100k indels at the *TET3* locus), consistent with previous report [37]. Notably, e*Nme2*-C.NR and the *IS*Dra2 exhibited significant junction enrichment at the U6-sgRNA position, in a zoomed-in view of this region, the enrichments occur mainly around the putative cut site at the spacer sites, suggesting their potential ability to cleave the U6-sgRNA region. Notably, *IS*Dra2 displayed U6-sgRNA enrichment at the *TET3* locus but not at the *TIGIT* locus, indicating site-specific variation of U6-sgRNA cleavage (Fig. 3d and Additional file 1: Fig. S4b).

Taken together, the TnpB system generates minor nucleotide insertions predominantly within 25 bp and produces plasmid/mtDNA insertions as other CRISPR systems.

### Evaluation of the editing specificity

**Fig. 4:**
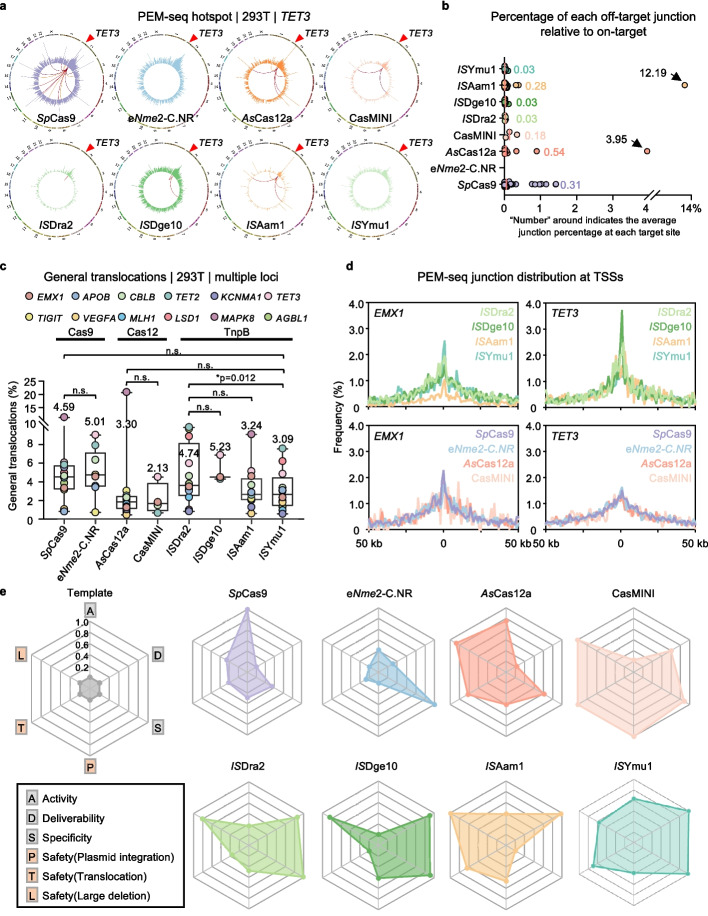
Statistics for translocations during gene editing with various nucleases. **a** Genome-wide translocation distribution patterns (circos plot) for indicated nucleases at *TET3* locus. The translocation signals (2-Mb bins) were plotted on a log scale. On-target cleavage sites are indicated by red arrowheads. The lines connecting the junctions indicate ON–OFF target translocations. **b** The percentage of off-target counts relative to the on-target counts at all identified off-target sites for these nucleases. The numbers indicate the average percentage of off-target counts relative to on-target counts across all off-target sites. Note that the translocation junctions within 100 bp of the identified off-target were included in corresponding off-target counts. **c** The translocation percentages for the indicated nucleases. The numbers indicate the average translocation percentages across all effective editing sites (Data are presented as mean ± SD). Paired two-tailed t-test, n.s., not significant, *p ≤ 0.05. **d** The distribution patterns of translocation junctions around TSSs at the *EMX1* or *TET3* locus. Translocations within the U6 and off-target regions of the genome are excluded from analysis. **e** The radar plots show the editing characteristics of various nucleases spanning six key dimensions. Activity scores were obtained by min–max normalization of the average editing efficiency corresponding to all enzymes; deliverability scores were obtained by calculating the delivery size of each nuclease (comprising protein size and gRNA length), then taking the reciprocal of this value, followed by min–max normalization; the specificity scores are obtained by min–max normalizing the proportion of on-target junctions (on-target indels/total editing events) for each nuclease; the specificity scored are obtained by min–max normalizing the value calculated by [1 – off-target junctions/on target junctions%] for each nuclease; the safety (plasmid integration) scores were obtained by min–max normalizing the value calculated by [1 – plasmid integrations%]; the safety(translocation) and safety(large deletion) scores were obtained by min–max normalizing the value calculated by [1 – general translocations%] and [1 – large deletions%] respectively

After extensively characterizing the events at the target site, we next sought to assess the off-target activities of these eight systems across all tested loci. The off-target DSBs during gene editing can be captured as chromosomal translocations via PEM-seq when they fused with on-target DSBs. We conducted a hotspot-finding analysis of identified translocation junctions to identify off-target sites for these eight systems across all tested loci (Fig. 4a). *IS*Aam1, *Sp*Cas9, and *IS*Dge10 induced cleavage at multiple off-target sites (49, 30, and 11, respectively) across these tested loci, while *IS*Ymu1 and *As*Cas12a had lower off-target frequencies, with 10 and 9 sites, respectively. e*Nme2*-C.NR, CasMINI, and *IS*Dra2 yielded the fewest off-target sites (0, 3, and 5, respectively) (Additional file 1: Fig. S5a). Taken the *TET3* site as an example, all eight systems achieved effective cleavage, however, *Sp*Cas9, *As*Cas12a, and *IS*Aam1 induced cleavage at multiple off-target sites (ranging from 7 to 21 sites), whereas e*Nme2*-C.NR and *IS*Ymu1 did not yield any detectable off-target sites (Fig. 4a and Additional file 1: Fig. S5a).

To measure the off-target strength, we calculated the ratio of off-target junctions to on-target edits junctions by counting the junctions within 100 bp around each target and off-target sites. Overall, these tested systems exhibited relatively weak off-target effects, with average off-target ratios ranging from 0.02% to 0.60% (Fig. 4b and Additional file 1: Fig. S5b). Specifically, *IS*Aam1 and *As*Cas12a showed strong off-target effects at the abovementioned *TET3* site, with off-target ratios reaching 12.19% and 3.95%, respectively, at the strongest off-target site (*TET3*: *IS*Aam1-OT1 and *As*Cas12a-OT1) (Fig. 4a, b and Additional file 1: Fig. S6). Sequence analysis of these two strong off-target sites revealed only three mismatches in the gRNA body, with one mismatch base located in the TAM/PAM-proximal region and the other two mismatch bases located at the nucleotide most distal from the TAM/PAM sequence (Additional file 1: Fig. S6).

To further validate these findings using an orthogonal method, we performed GUIDE-seq analysis at the *TET3* site for four representative editors (*Sp*Cas9, *As*Cas12a, *IS*Aam1, and *IS*Ymu1) [41]. GUIDE-seq detected 16 off-target sites for *Sp*Cas9, 14 of which overlapped with the 21 sites identified by PEM-seq. GUIDE-seq uniquely identified 2 sites, while PEM-seq independently detected 7 additional weak off-target sites (OT10, OT11, OT12, OT16, OT19, OT20, and OT21). For *As*Cas12a, all 6 off-target sites found by GUIDE-seq were also captured by PEM-seq (which detected 8 sites in total, with OT6 and OT7 unique to PEM-seq). For *IS*Aam1, GUIDE-seq identified 11 off-targets, 5 of which overlapped with PEM-seq results. GUIDE-seq uniquely detected 6 sites, and PEM-seq independently identified 2 weak off-targets (OT6 and OT7). Most notably, for *IS*Ymu1, GUIDE-seq detected no off-target sites, consistent with PEM-seq which registered only one very weak potential off-target signal (off-target ratio = 0.01%). These results strongly corroborate the specificity profiles revealed by PEM-seq (Additional file 1: Figs. S6 and S7a, b).

Overall, *IS*Dra2 and *IS*Ymu1 TnpB systems may possess greater specificity than Cas9 or Cas12a tools, while displayed comparable specificity to CasMINI or e*Nme2*-C.NR tools.

### Evaluation of the genomic structural variations induced by gene editing

In addition to off-target damages, genome-wide collateral damages can also form chromosomal translocations with the on-target DSBs during gene editing. These general and unpredictable chromosomal translocations are deleterious byproducts of the gene editing processes, potentially threatening the genome integrity. We analyzed the general translocations induced by these eight systems by calculating the total number of translocations subtracting off-target DSBs-involved translocations. The levels of TnpB-induced general translocations ranged from an average of 3.09% for *IS*Ymu1 to 5.23% for *IS*Dge10, compared to 4.59% for *Sp*Cas9 and 5.01% for e*Nme2*-C.NR. Notably, the translocation levels for Cas12 systems were even lower, with *As*Cas12a at 3.30% and CasMINI at 2.13% (Fig. 4c). Consistent with previous findings [37], our analysis of the general distribution of translocation revealed a predominant enrichment at transcription start sites (TSSs) (Fig. 4d), suggesting that active transcription, as an editing-independent factor, primarily contributes to collateral DSBs during gene editing.

## Discussion

Compare to the Cas9 family, Cas12 is a much more diverse family. In recent years, multiple Cas12 subtypes have been explored and developed into gene editing tools [18–28]. The discovery of the evolutionary ancestor of Cas12 nuclease, TnpB, further added to the diversity of gene editing tools [29]. In particular, TnpB represents the most compact gene editing tool currently available. To guide the selection of the most suitable system for gene editing, we systematically evaluated the editing characteristics of four TnpB systems in comparison with the widely used Cas9 and Cas12a, as well as the recently developed miniature Cas12f. Based on collected data on editing activity, specificity, and safety, we visualize the relative editing performance of these eight systems across six dimensions: activity (A), deliverability (D), specificity (S), and safety profile based on the frequency of plasmid integration tolerance (P), translocation tolerance (T), or large deletion tolerance (L) (Fig. 4e). Although Cas9 exhibits the highest editing activity, it has poor deliverability, specificity, and safety. Among the Cas12 family, CasMINI performs relatively well in five dimensions but has poor editing activity, highlighting the need to enhance its activity through modification or target site pre-screening, as noted in previous study [42]. Among the TnpB family, our newly developed *IS*Ymu1 TnpB demonstrates favorable properties across all dimensions, making it one of the preferred choices for a miniature editing system. This conclusion is further supported by a recent study by Weiss et al., which systematically evaluated three TnpB editors (*IS*Dra2, *IS*Ymu1, and *IS*Aam1) and reported that *IS*Ymu1 demonstrated the highest average editing efficiency across multiple tested sites in Arabidopsis protoplasts, while whole-genome sequencing revealed minimal background mutations, reforcing its high specificity [43].

Compared to the 2–3 nt PAM requirement of the Cas9 and Cas12 systems, TnpBs have a 4–5 nt TAM requirement. On one hand, a relative long TAM sequence likely contributes to the specificity of targeted editing. Conversely, longer TAM imposes accessibility constraints for applications requiring editing at precise position. Directed evolution guided protein engineering has emerged as a common approach to shorten the PAM sequence requirement of CRISPR-Cas systems [17, 44–47]. A similar strategy could be employed to engineer TnpB tools with shorter TAM sequence, thereby expanding their targeting accessibility. However, the shortening of PAM often comes at the cost of compromised enzymatic activity or editing specificity [48]. Based on our previously established framework for annotating and predicting active TnpB systems in prokaryotic genomes [35], we are currently conducting a large-scale screening of TnpBs with different predicted TAMs in human cell lines. In addition, recent study identified multiple active TnpBs with CG-rich TAMs from IS607 family [49, 50]. Thus, a tool box composed of many TnpB genome editors with different TAM preference will satisfy both target specificity and accessibility.

It is worth noting that the versatility of TnpB nucleases extends beyond their established role in genome editing. Recent study highlighted their potential role as gene regulation effectors [51]. Undoubtedly, additional effector functions of TnpB domestication will be discovered through further research. In addition, the miniature size of TnpB proteins makes them optimal DNA binding modules. By fusing TnpB with various functional domains, tools such as miniature base editor, prime editor, and epigenome editor will be developed. This study only characterized the specificity of generating targeted DSB by TnpBs, while future efforts are needed to evaluate their specificity of binding to targeted DNA. Future validation in practical settings, along with large-scale profiling across diverse genomic contexts and cell types, will be crucial to further refine our understanding of TnpB editor performance and their therapeutic potential.

## Conclusions

Taken together, by comprehensively analyzing genome editing outcomes, we characterized TnpB systems for genome editing and identified *IS*Ymu1 TnpB as an optimal miniature RNA-guided genome editors with balanced performance, highlighting its potential for therapeutic applications.

## Methods

### Plasmid construction

Various TnpB systems and Cas nucleases were cloned into the px330 plasmid vector (Addgene ID 42230) with mCherry marker gene for cell sorting in the PEM-seq assay. The vector information of different TnpB systems and Cas nucleases were provided in Source data. All the tested sgRNAs are listed in the Additional file 2: Table S1.

### Cell culture and plasmid transfection

HEK293T cells were obtained from the American Type Culture Collection (ATCC), which were confirmed to be free of mycoplasma contamination. HEK293T cells were cultured in Dulbecco′s modified Eagle′s medium (Corning) with 10% Fetal Bovine Serum (FBS, Gibco) and 1% Penicillin–Streptomycin (PS, Gibco) at 37 °C with 5% CO_2_. HEK293T cells were seeded in 6-well plates at a density of one million cells per well. They were then transfected with 3 μg of the pX330-TnpB/Cas nuclease-P2A-mCherry plasmid using 6 μl of Lipofectamine 2000 transfection reagent (Invitrogen) per well, resulting in a total of three million cells being transfected by each nuclease. TnpB/Cas nuclease-transfected cells were harvested 72 h post-transfection with an Aria Fusion flow cytometry sorter based on mCherry expression. Genomic DNA was then extracted for PEM-seq library construction.

### PEM-seq assay

The fundamental process of constructing a PEM-seq library involves a series of steps and typically requires 20 μg of genomic DNA [36]. First, edited cells are harvested, and genomic DNA is extracted by cell lysis and ethanol precipitation, followed by fragmentation through sonication. Subsequently, biotinylated primers designed within 150 bp from the cut site are utilized for linear amplification of target products, followed by removal of the biotinylated primers. The biotinylated amplification products are then enriched with streptavidin C1 beads and ligated with “bridge adapters” containing random molecular barcodes (RMBs). Nested PCR is then performed for a second round of exponential amplification of target product sequences, followed by the addition of Illumina sequencing adapters through tagged PCR and high-throughput sequencing.

The bio-primers and nested primers used in this study are documented in Additional file 2: Table S1.

### PEM-Q analysis

The sequencing data were analyzed using the PEM-Q pipeline described above. In general, the editing products containing insertions, deletions, and translocations can be identified by the PEM-Q pipeline. Editing efficiency is defined as the ratio of the number of indels and translocations to the total number of identified sequencing reads. To ensure a fair comparison of editing outcomes and specificity across nucleases, all event frequencies were normalized to on-target activity. Specifically, the frequencies of abnormal events (e.g., large deletions, translocations, and plasmid integrations) are reported as proportions relative to editing events, not as a fraction of total sequencing reads. Deletions were clarified as small deletions (≤ 100 bp) and large deletions (> 100 bp). Insertions consisted of small insertions (≤ 20 bp) and large insertions (> 20 bp). For off-target analysis, translocation hotspot sequences that closely resembling the target site (≤ 8 nt mismatches containing PAM sequences) and with putative cut-site junction patterns were regarded as off-target sites. Furthermore, translocation junctions located within 100 bp around the identified off-target sites were considered off-target junctions. General translocations excluded junctions within 500 kb upstream and downstream of the target site, as well as off-target translocations.

For an elaborate understanding of the bioinformatic analysis tool used, please refer to the previous investigation of the PEM-Q pipeline [37].

### Target sequencing analysis

NGS library construction was carried out in a two-step PCR protocol: Step 1 (Target Amplification): 0.5 μg of genomic DNA was amplified for 20 cycles in a 25-μL reaction. Step 2 (Barcoding): 1 μL of the first-round PCR product was used as the template for an 11-cycle amplification to incorporate sample-indexing barcodes. Amplicons underwent illumine Hiseq sequencing followed by CRISPResso 2 (version 2.3.0) analysis with default parameters(–ignore_substitutions –amplicon_min_alignment_score 70). The gRNA sequence and NGS primers designed in this study are documented in Additional file 2: Tables S2 and S3.

### GUIDE-seq analysis

HEK293T cells (4 × 10^5^) were transfected with 2 μg of CRISPR expression plasmid and 20 pmol of pre-annealed GUIDE-seq oligodeoxynucleotides using the Lonza 4D-Nucleofector® system, and subsequently cultured in 6-well plates. Genomic DNA was extracted 72 h post-transfection. Target regions were amplified by nested PCR, and the resulting products were purified by gel-based size selection for deep sequencing [41].

### Statistical analysis

All studies of biological phenomena were conducted with at least seven sample sizes. Data are presented as mean ± SD, with detailed information on sample sizes provided in the figure legends. One-way ANOVA with Geisser-Greenhouse correction statistical analysis was performed on at least three biologically independent experiments using GraphPad Prism8, and *p* < 0.05 was considered significant. No statistical method was used to determine sample size. No data were excluded from analyses, and the experiments were not randomized. Furthermore, the investigators were not blinded to allocation during both the experiments and the outcome assessment.

## Supplementary Information

Additional file 1: Figure S1. gRNA design for all tested target sites. Figure S2. The cleavage mechanism detected by PEM-seq. Figure S3. Typical deletional products of different nucleases. Figure S4. The insertion length distribution and plasmid junction distribution of different nucleases. Figure S5. PEM-seq detected genome-wide editing off-targets of various nucleases. Figure S6. Sequence alignments and reads counts of PEM-seq detected genome-wide editing off-targets of various nucleases at TET3 locus. Figure S7. GUIDE-seq detected genome-wide editing off-targets of different nucleases.

Additional file 2: Table S1. Bio-primer, Red-primer, and gRNA sequences used in PEM-seq assay. Table S2. gRNA sequences and NGS primers design in Target-sequencing assay. Table S3. gRNA sequences and NGS primers design in Target-sequencing assay.

Additional file 3: Table S4. Key data resource table used to generate figures.

## Data Availability

All sequencing data presented in this study have been deposited in the NCBI Gene Expression Omnibus (GEO) database under accession code GSE264389 [52]. Support code for PEM-seq analysis are available at GitHub website [53] and zenodo [54]. Genome editing vectors are available at Wekwikgene (https://wekwikgene.wllsb.edu.cn/labs/a0c6fde2-479d-4ead-b008-3672a6ddf8a4) with ID number (0002333, 0002334, 0002335, 0002336, 0002337, 0002338, 00023339 and 0002340). Data used to generate figures is available in Additional file 3: Table S4.

## References

[1] Cong L, Ran FA, Cox D, Lin S, Barretto R, Habib N, et al. Multiplex genome engineering using CRISPR/Cas systems. Science. 2013;339:819–23. 23287718 10.1126/science.1231143 PMC3795411

[2] Mali P, Yang L, Esvelt KM, Aach J, Guell M, DiCarlo JE, et al. RNA-guided human genome engineering via Cas9. Science. 2013;339:823–6. 23287722 10.1126/science.1232033 PMC3712628

[3] Zetsche B, Gootenberg JS, Abudayyeh OO, Slaymaker IM, Makarova KS, Essletzbichler P, et al. Cpf1 is a single RNA-guided endonuclease of a class 2 CRISPR-Cas system. Cell. 2015;163:759–71. 26422227 10.1016/j.cell.2015.09.038 PMC4638220

[4] Wang H, Yang H, Shivalila CS, Dawlaty MM, Cheng AW, Zhang F, et al. One-step generation of mice carrying mutations in multiple genes by CRISPR/Cas-mediated genome engineering. Cell. 2013;153:910–8. 23643243 10.1016/j.cell.2013.04.025 PMC3969854

[5] Chavez M, Chen X, Finn PB, Qi LS. Advances in CRISPR therapeutics. Nat Rev Nephrol. 2023;19:9–22. 36280707 10.1038/s41581-022-00636-2 PMC9589773

[6] Gillmore JD, Gane E, Taubel J, Kao J, Fontana M, Maitland ML, et al. CRISPR-Cas9 In Vivo Gene Editing for Transthyretin Amyloidosis. N Engl J Med. 2021;385:493–502. 34215024 10.1056/NEJMoa2107454

[7] Ledford H. CRISPR 2.0: a new wave of gene editors heads for clinical trials. Nature. 2023;624:234–5. 38062143 10.1038/d41586-023-03797-7

[8] Sheridan C. The world’s first CRISPR therapy is approved: who will receive it? Nat Biotechnol. 2024;42:3–4. 37989785 10.1038/d41587-023-00016-6

[9] Wang D, Zhang F, Gao G. CRISPR-based therapeutic genome editing: strategies and in vivo delivery by AAV vectors. Cell. 2020;181:136–50. 32243786 10.1016/j.cell.2020.03.023 PMC7236621

[10] Colella P, Ronzitti G, Mingozzi F. Emerging issues in AAV-mediated in vivo gene therapy. Mol Ther Methods Clin Dev. 2018;8:87–104. 29326962 10.1016/j.omtm.2017.11.007 PMC5758940

[11] Komor AC, Kim YB, Packer MS, Zuris JA, Liu DR. Programmable editing of a target base in genomic DNA without double-stranded DNA cleavage. Nature. 2016;533:420–4. 27096365 10.1038/nature17946 PMC4873371

[12] Anzalone AV, Randolph PB, Davis JR, Sousa AA, Koblan LW, Levy JM, et al. Search-and-replace genome editing without double-strand breaks or donor DNA. Nature. 2019;576:149–57. 31634902 10.1038/s41586-019-1711-4 PMC6907074

[13] Levy JM, Yeh WH, Pendse N, Davis JR, Hennessey E, Butcher R, et al. Cytosine and adenine base editing of the brain, liver, retina, heart and skeletal muscle of mice via adeno-associated viruses. Nat Biomed Eng. 2020;4:97–110. 31937940 10.1038/s41551-019-0501-5 PMC6980783

[14] Davis JR, Banskota S, Levy JM, Newby GA, Wang X, Anzalone AV, et al. Efficient prime editing in mouse brain, liver and heart with dual AAVs. Nat Biotechnol. 2024;42(2):253–64. 37142705 10.1038/s41587-023-01758-z PMC10869272

[15] Ran FA, Cong L, Yan WX, Scott DA, Gootenberg JS, Kriz AJ, et al. In vivo genome editing using *Staphylococcus aureus* Cas9. Nature. 2015;520:186–91. 25830891 10.1038/nature14299 PMC4393360

[16] Kim E, Koo T, Park SW, Kim D, Kim K, Cho HY, et al. In vivo genome editing with a small Cas9 orthologue derived from *Campylobacter jejuni*. Nat Commun. 2017;8:14500. 28220790 10.1038/ncomms14500 PMC5473640

[17] Huang TP, Heins ZJ, Miller SM, Wong BG, Balivada PA, Wang T, et al. High-throughput continuous evolution of compact Cas9 variants targeting single-nucleotide-pyrimidine PAMs. Nat Biotechnol. 2023;41:96–107. 36076084 10.1038/s41587-022-01410-2 PMC9849140

[18] Liu JJ, Orlova N, Oakes BL, Ma E, Spinner HB, Baney KLM, et al. CasX enzymes comprise a distinct family of RNA-guided genome editors. Nature. 2019;566:218–23. 30718774 10.1038/s41586-019-0908-x PMC6662743

[19] Tsuchida CA, Zhang S, Doost MS, Zhao Y, Wang J, O’Brien E, et al. Chimeric CRISPR-CasX enzymes and guide RNAs for improved genome editing activity. Mol Cell. 2022;82(1199–1209):e1196. 10.1016/j.molcel.2022.02.002 PMC9189900 35219382

[20] Harrington LB, Burstein D, Chen JS, Paez-Espino D, Ma E, Witte IP, et al. Programmed DNA destruction by miniature CRISPR-Cas14 enzymes. Science. 2018;362:839–42. 30337455 10.1126/science.aav4294 PMC6659742

[21] Karvelis T, Bigelyte G, Young JK, Hou Z, Zedaveinyte R, Budre K, et al. Pam recognition by miniature CRISPR-Cas12f nucleases triggers programmable double-stranded DNA target cleavage. Nucleic Acids Res. 2020;48:5016–23. 32246713 10.1093/nar/gkaa208 PMC7229846

[22] Kim DY, Lee JM, Moon SB, Chin HJ, Park S, Lim Y, et al. Efficient CRISPR editing with a hypercompact Cas12f1 and engineered guide RNAs delivered by adeno-associated virus. Nat Biotechnol. 2022;40:94–102. 34475560 10.1038/s41587-021-01009-z PMC8763643

[23] Wu Z, Zhang Y, Yu H, Pan D, Wang Y, Wang Y, et al. Programmed genome editing by a miniature CRISPR-Cas12f nuclease. Nat Chem Biol. 2021;17:1132–8. 34475565 10.1038/s41589-021-00868-6

[24] Xu X, Chemparathy A, Zeng L, Kempton HR, Shang S, Nakamura M, et al. Engineered miniature CRISPR-Cas system for mammalian genome regulation and editing. Mol Cell. 2021;81(4333–4345):e4334. 10.1016/j.molcel.2021.08.008 34480847

[25] Pausch P, Al-Shayeb B, Bisom-Rapp E, Tsuchida CA, Li Z, Cress BF, et al. Crispr-CasPhi from huge phages is a hypercompact genome editor. Science. 2020;369:333–7. 32675376 10.1126/science.abb1400 PMC8207990

[26] Pausch P, Soczek KM, Herbst DA, Tsuchida CA, Al-Shayeb B, Banfield JF, et al. DNA interference states of the hypercompact CRISPR-CasPhi effector. Nat Struct Mol Biol. 2021;28:652–61. 34381246 10.1038/s41594-021-00632-3 PMC8496406

[27] Sun A, Li CP, Chen Z, Zhang S, Li DY, Yang Y, Li LQ, Zhao Y, Wang K, Li Z, et al. The compact Caspi (Cas12l) ‘bracelet’ provides a unique structural platform for DNA manipulation. Cell Res. 2023;33(3):229–44. 10.1038/s41422-022-00771-2 PMC9977741 36650285

[28] Chen W, Ma J, Wu Z, Wang Z, Zhang H, Fu W, et al. Cas12n nucleases, early evolutionary intermediates of type V CRISPR, comprise a distinct family of miniature genome editors. Mol Cell. 2023;83(2768–2780):e2766. 10.1016/j.molcel.2023.06.014 37402371

[29] Karvelis T, Druteika G, Bigelyte G, Budre K, Zedaveinyte R, Silanskas A, et al. Transposon-associated TnpB is a programmable RNA-guided DNA endonuclease. Nature. 2021;599:692–6. 34619744 10.1038/s41586-021-04058-1 PMC8612924

[30] Nakagawa R, Hirano H, Omura SN, Nety S, Kannan S, Altae-Tran H, et al. Cryo-EM structure of the transposon-associated TnpB enzyme. Nature. 2023;616:390–7. 37020030 10.1038/s41586-023-05933-9 PMC10097598

[31] Sasnauskas G, Tamulaitiene G, Druteika G, Carabias A, Silanskas A, Kazlauskas D, et al. Tnpb structure reveals minimal functional core of Cas12 nuclease family. Nature. 2023;616:384–9. 37020015 10.1038/s41586-023-05826-x

[32] Schuler G, Hu C, Ke A. Structural basis for RNA-guided DNA cleavage by IscB-omegaRNA and mechanistic comparison with Cas9. Science. 2022;376:1476–81. 35617371 10.1126/science.abq7220 PMC10041819

[33] Li Z, Guo R, Sun X, Li G, Shao Z, Huo X, et al. Engineering a transposon-associated TnpB-omegaRNA system for efficient gene editing and phenotypic correction of a tyrosinaemia mouse model. Nat Commun. 2024;15:831. 38280857 10.1038/s41467-024-45197-z PMC10821889

[34] Wang M, Sun Z, Liu Y, Yin P, Liang C, Tan L, et al. Hypercompact TnpB and truncated TnpB systems enable efficient genome editing in vitro and in vivo. Cell Discov. 2024;10:31. 38503726 10.1038/s41421-023-00645-w PMC10951260

[35] Xiang G, Li Y, Sun J, Huo Y, Cao S, Cao Y, et al. Evolutionary mining and functional characterization of TnpB nucleases identify efficient miniature genome editors. Nat Biotechnol. 2023;42(5):745–57. 10.1038/s41587-023-01857-x 37386294

[36] Yin J, Liu M, Liu Y, Wu J, Gan T, Zhang W, et al. Optimizing genome editing strategy by primer-extension-mediated sequencing. Cell Discov. 2019;5:18. 30937179 10.1038/s41421-019-0088-8 PMC6434046

[37] Liu M, Zhang W, Xin C, Yin J, Shang Y, Ai C, et al. Global detection of DNA repair outcomes induced by CRISPR-Cas9. Nucleic Acids Res. 2021;49:8732–42. 34365511 10.1093/nar/gkab686 PMC8421148

[38] Liu Y, Yin J, Gan T, Liu M, Xin C, Zhang W, et al. PEM-seq comprehensively quantifies DNA repair outcomes during gene-editing and DSB repair. STAR Protoc. 2022;3:101088. 35462794 10.1016/j.xpro.2021.101088 PMC9019705

[39] Jiang F, Doudna JA. CRISPR-Cas9 structures and mechanisms. Annu Rev Biophys. 2017;46:505–29. 28375731 10.1146/annurev-biophys-062215-010822

[40] Wu J, Liu Y, Ou L, Gan T, Zhangding Z, Yuan S, et al. Transfer of mitochondrial DNA into the nuclear genome during induced DNA breaks. Nat Commun. 2024;15:9438. 39487167 10.1038/s41467-024-53806-0 PMC11530683

[41] Tsai SQ, Zheng Z, Nguyen NT, Liebers M, Topkar VV, Thapar V, et al. GUIDE-seq enables genome-wide profiling of off-target cleavage by CRISPR-Cas nucleases. Nat Biotechnol. 2015;33:187–97. 25513782 10.1038/nbt.3117 PMC4320685

[42] Xin C, Yin J, Yuan S, Ou L, Liu M, Zhang W, et al. Comprehensive assessment of miniature CRISPR-Cas12f nucleases for gene disruption. Nat Commun. 2022;13:5623. 36153319 10.1038/s41467-022-33346-1 PMC9509373

[43] Weiss T, Kamalu M, Shi H, Li Z, Amerasekera J, Zhong Z, et al. Viral delivery of an RNA-guided genome editor for transgene-free germline editing in Arabidopsis. Nat Plants. 2025;11:967–76. 40263581 10.1038/s41477-025-01989-9 PMC12095077

[44] Walton RT, Christie KA, Whittaker MN, Kleinstiver BP. Unconstrained genome targeting with near-PAMless engineered CRISPR-Cas9 variants. Science. 2020;368:290–6. 32217751 10.1126/science.aba8853 PMC7297043

[45] Chatterjee P, Jakimo N, Lee J, Amrani N, Rodriguez T, Koseki SRT, et al. An engineered ScCas9 with broad PAM range and high specificity and activity. Nat Biotechnol. 2020;38:1154–8. 32393822 10.1038/s41587-020-0517-0

[46] Miller SM, Wang T, Randolph PB, Arbab M, Shen MW, Huang TP, et al. Continuous evolution of SpCas9 variants compatible with non-G PAMs. Nat Biotechnol. 2020;38:471–81. 32042170 10.1038/s41587-020-0412-8 PMC7145744

[47] Schmidheini L, Mathis N, Marquart KF, Rothgangl T, Kissling L, Bock D, et al. Continuous directed evolution of a compact CjCas9 variant with broad PAM compatibility. Nat Chem Biol. 2024;20:333–43. 37735239 10.1038/s41589-023-01427-x PMC7616171

[48] Zhang W, Yin J, Zhang-Ding Z, Xin C, Liu M, Wang Y, et al. In-depth assessment of the PAM compatibility and editing activities of Cas9 variants. Nucleic Acids Res. 2021;49(15):8785–95. 10.1093/nar/gkab507 PMC8421146 34133740

[49] Ren K, Zhou F, Zhang F, Yin M, Zhu Y, Wang S, et al. Discovery and structural mechanism of DNA endonucleases guided by RAGATH-18-derived RNAs. Cell Res. 2024;34:370–85. 38575718 10.1038/s41422-024-00952-1 PMC11061315

[50] Yang H, Patel DJ. IS607 TnpB is a hypercompact RNA-guided DNA endonuclease. Cell Res. 2024;34:473–4. 38744980 10.1038/s41422-024-00973-w PMC11217443

[51] Wiegand T, Hoffmann FT, Walker MWG, Tang S, Richard E, Le HC, et al. TnpB homologues exapted from transposons are RNA-guided transcription factors. Nature. 2024;631:439–48. 38926585 10.1038/s41586-024-07598-4 PMC11702177

[52] Xin C XG, Cao S, Wang Y, Hu J, Wang H. Comprehensive assessment of activity, specificity, and safety of hypercompact TnpB systems for gene editing. GSE264389. Gene Expression Omnibus (GEO), https://www.ncbi.nlm.nih.gov/geo/query/acc.cgi?acc=GSE264389. 2025. 10.1186/s13059-026-03949-8 PMC12908284 41566394

[53] Liu M ZW, Xin C, Hu J. Global detection of DNA repair outcomes induced by CRISPR–Cas9, https://github.com/JiazhiHuLab/PEM-Q. 2021. 10.1093/nar/gkab686 PMC8421148 34365511

[54] Liu M. PEM-Q(v1.0.0).Zenodo, 10.5281/zenodo.18176124. 2026.

