# Supplementary material for: Comprehensive assessment of activity, specificity, and safety of hypercompact TnpB systems for gene editing
Source: Genome Biol. 2026 Jan 21;27:39. doi: 10.1186/s13059-026-03949-8 (PMC12908284; doi:10.1186/s13059-026-03949-8)
Supplement: Supplementary file 1 — Additional file 1: Figure S1. gRNA design for all tested target sites. Figure S2. The cleavage mechanism detected by PEM-seq. Figure S3. Typical deletional products of different nucleases. Figure S4. The insertion length distribution and plasmid junction distribution of different nucleases. Figure S5. PEM-seq detected genome-wide editing off-targets of various nucleases. Figure S6. Sequence alignments and reads counts of PEM-seq detected genome-wide editing off-targets of various nucleases at TET3 locus. Figure S7. GUIDE-seq detected genome-wide editing off-targets of different nucleases. [file 13059_2026_3949_MOESM1_ESM.pdf]

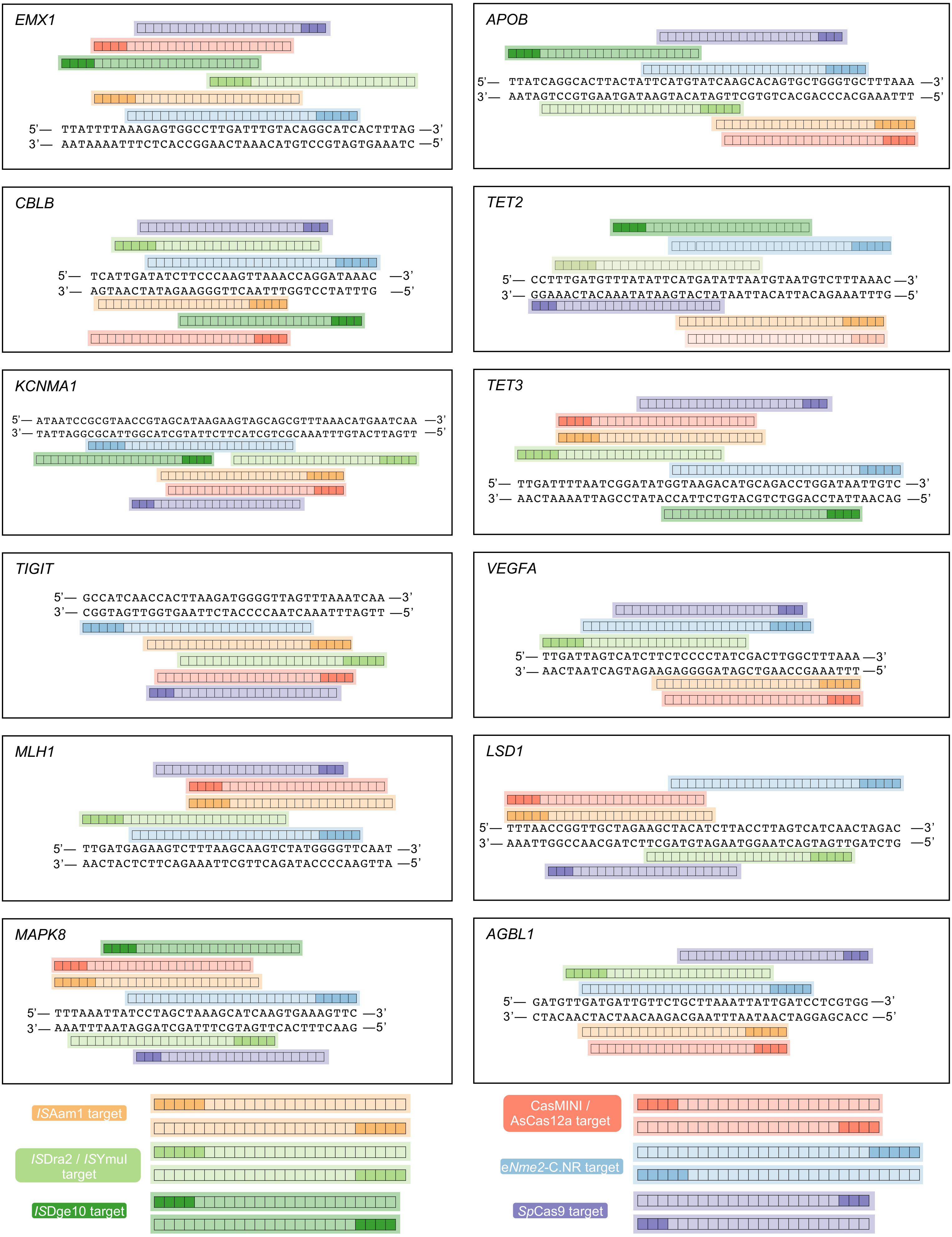

Figure S1. gRNA design for all tested target sites. Related to Figure 1.

Figure S1. gRNA design for all tested target sites. Related to Figure 1.
Overview of all tested target sites designed to compare eight representative TnpB/Cas12/Cas9 nucleases: *IS*Aam1, *IS*Ymu1, *IS*Dra2, *IS*Dge10, CasMINI, *As*Cas12a, e*Nme2*-C.NR, and *Sp*Cas9. Please note that *TIGIT*, *VEGFA*, *MLH1*, *LSD1*, and *AGBL1* loci do not have target sites for *IS*Dge10 due to the lack of a suitable PAM. For each target site, the corresponding gRNA design's position was annotated as either above or below the target sequence, depending on the distribution of target DNA on the upper and lower strands.

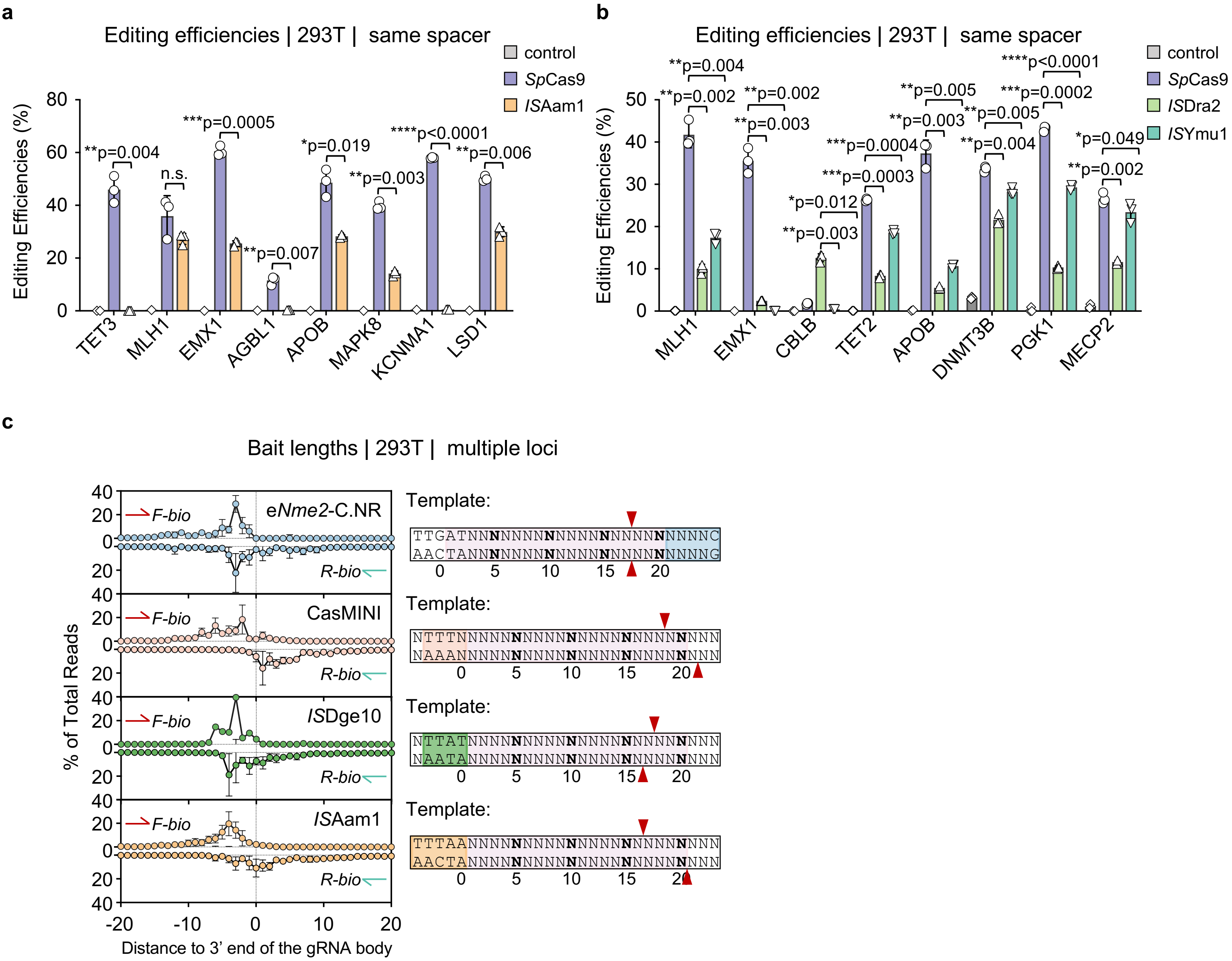

Figure S2. The cleavage mechanism detected by PEM-seq. Related to Figure 1.
(**a-b**) NGS detect the editing efficiencies across distinct genome loci. Data are shown as mean ± SD for n = 3 repeats. Paired two-tailed t-test, n.s., not significant, $*p \leq 0.05$, $**p \leq 0.01$, $***p \leq 0.001$, $****p \leq 0.0001$.
(**c**) Left: the location distribution and frequency of bait broken ends across all effective editing sites for e*Nme2*-C.NR, CasMINI, *IS*Dge10, and *IS*Aam1. The bait broken ends are categorized as target-strand broken ends and non-target-strand broken ends based on the design direction of the Bio-primer. The horizontal axis indicates specific positions, with black dotted lines marking the 3' end of the gRNA body. The vertical axis represents the probability of bait broken ends mapping at that exact location. Right: schematic diagrams illustrate speculated double-strand DNA cleavage patterns induced by specific nucleases, inferred from the distribution of bait broken ends. The major cleavage sites are indicated by red arrowheads, while the PAM sequences of various nucleases are highlighted with different colors.

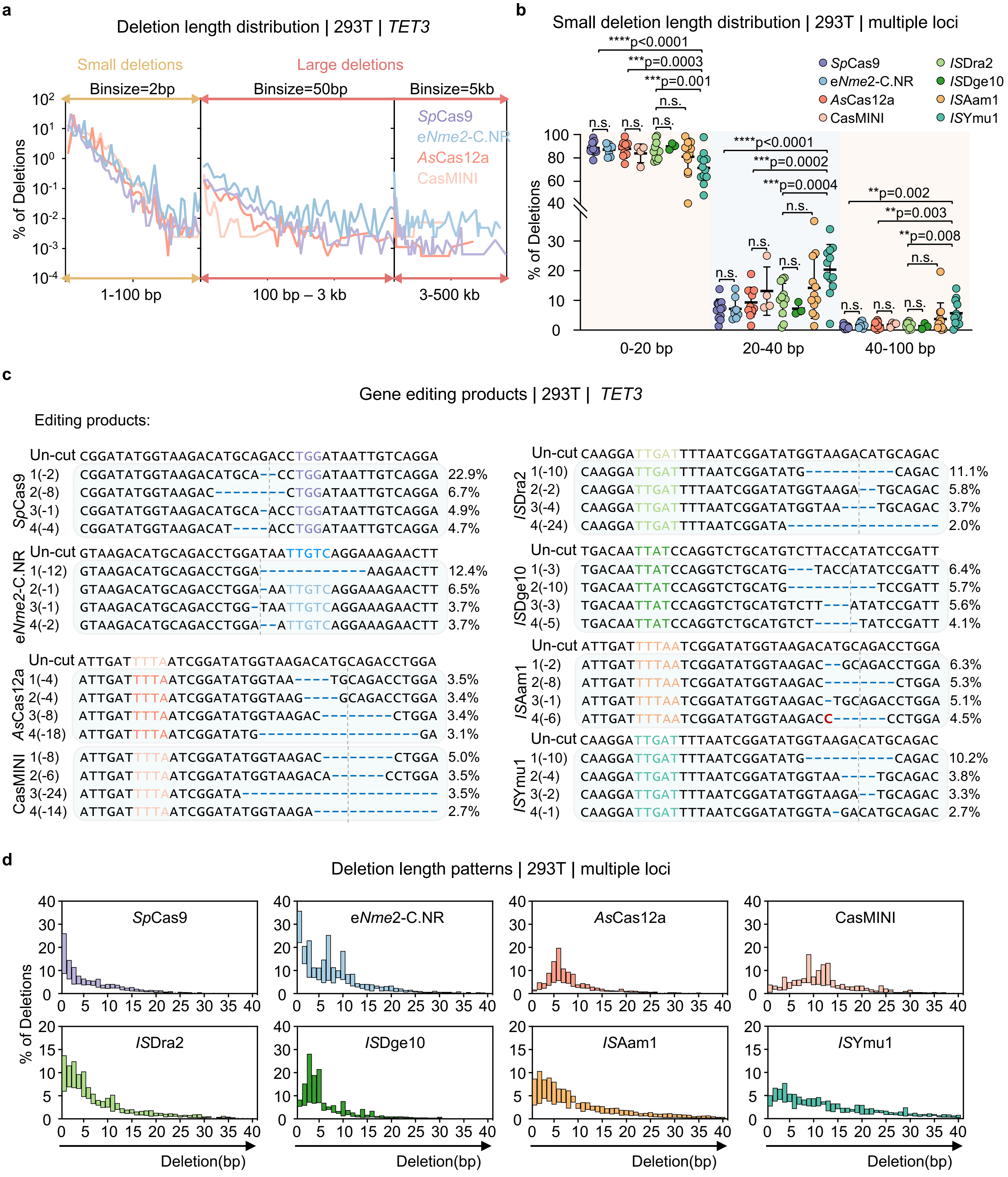

Figure S3. Typical deletional products of different nucleases. Related to Figure 2.

Figure S3. Typical deletional products of different nucleases. Related to Figure 2.
(**a**) Line chart showing the distribution pattern of deletion junctions for *Sp*Cas9, e*Nme2*-C.NR, *As*Cas12a, and CasMINI nucleases at the *TET3* locus in HEK293T cells. Total deletions were categorized into small deletions (<= 100 bp, in yellow) and large deletions (> 100 bp, in red), and further subdivided into 3 regions: junctions within 100 bp from the cut site; junctions ranging from 100 bp to 3 kb downstream from the cut site; and junctions ranging from 3 kb to 500 kb downstream from the cut site. Please note that bin sizes of 2 bp, 50 bp, and 5 kb were applied to these three regions, respectively.
(**b**) Summary scattered plots showing the length distribution of small deletions for all effective editing loci as detected by PEM-seq in HEK293T cells, where the vertical axis represents the percentage of indicated deletions relative to total deletions and the columns represent a subdivision into specific lengths. Paired two-tailed t-test, n.s., not significant, $^{**}p \leq 0.01$, $^{***}p \leq 0.001$, $^{****}p \leq 0.0001$.
(**c**) The top 4 most common editing products generated by the indicated nucleases at the *TET3* locus as detected by PEM-seq in HEK293T cells. The un-cut target sequences of each nuclease are shown at the top of the corresponding group, with the PAM sequences highlighted in different colors. The deleted nucleotides were marked with dashes and the inserted nucleotides were marked in red. The predicted cut sites were marked by the vertical dotted line and the mutation frequencies of corresponding editing events are listed on the right.
(**d**) The length and occurrence frequency information of deletions within a 40 bp range generated by the indicated nucleases at all effective editing loci. The vertical axis indicates the average ratio distribution across all loci, representing the number of deletion fragments with the specified length relative to the total number of deletion events.

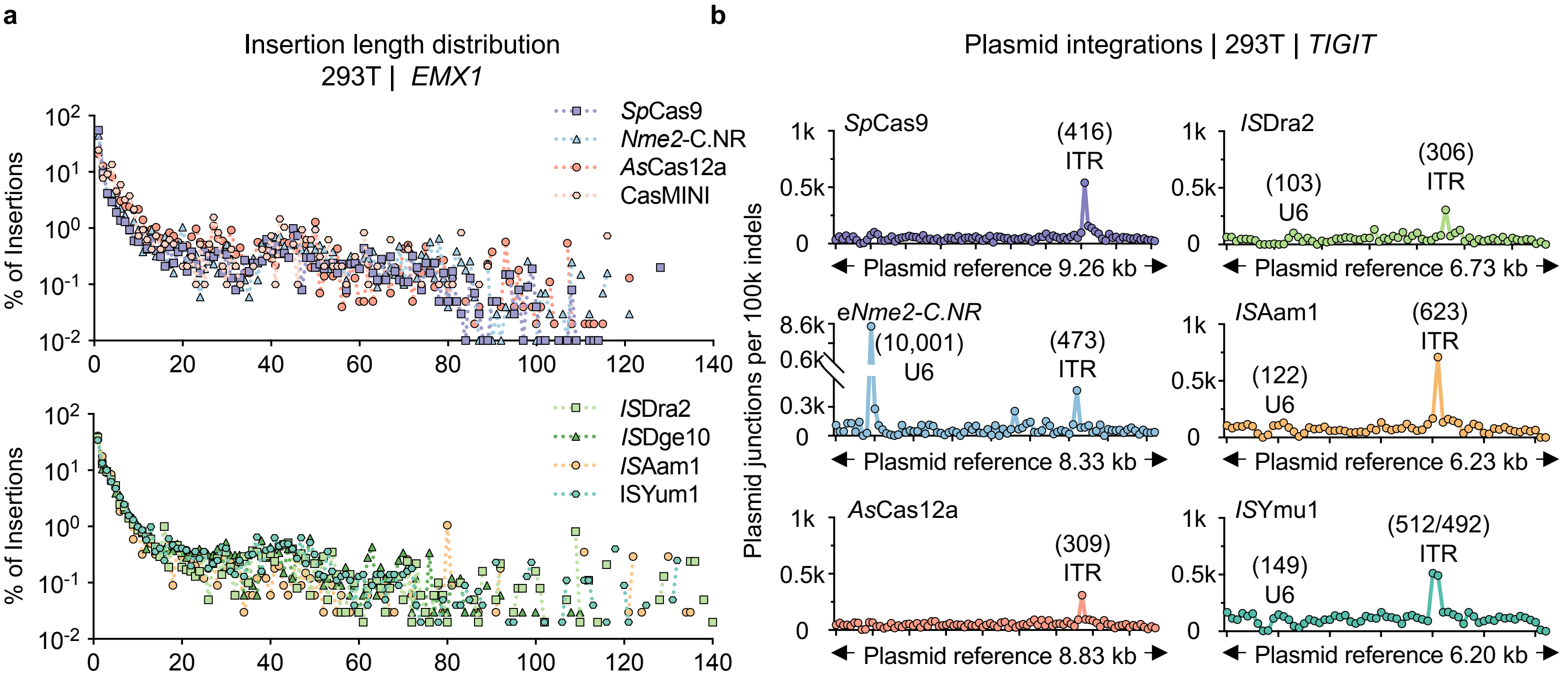

Figure S4. The insertion length distribution and plasmid junction distribution of different nucleases. Related to Figure 3.

(**a**) Line graph showing the distribution pattern of insertion junctions for TnpB and CRISPR tools at the *EMX1* locus in HEK293T cells. The horizontal axis represents the insertion length, and the vertical axis represents the proportion of insertions that occur exactly at the indicated location relative to the total number of insertions, presented on a logarithmic scale.

(**b**) The mapping position distribution of vector integration junctions across the respective plasmids for the different Cas nucleases at the *TIGIT* locus as detected by PEM-seq. The vertical axis represents the junction numbers of plasmid integrations that occur exactly at the indicated position when down-sampled to 100k indels. K means thousands. The bin size of the plasmid sequence is 100 bp. Junction numbers for the U6-sgRNA region and Adeno-associated virus (AAV) inverted repeat (ITR) regions are marked above separately.

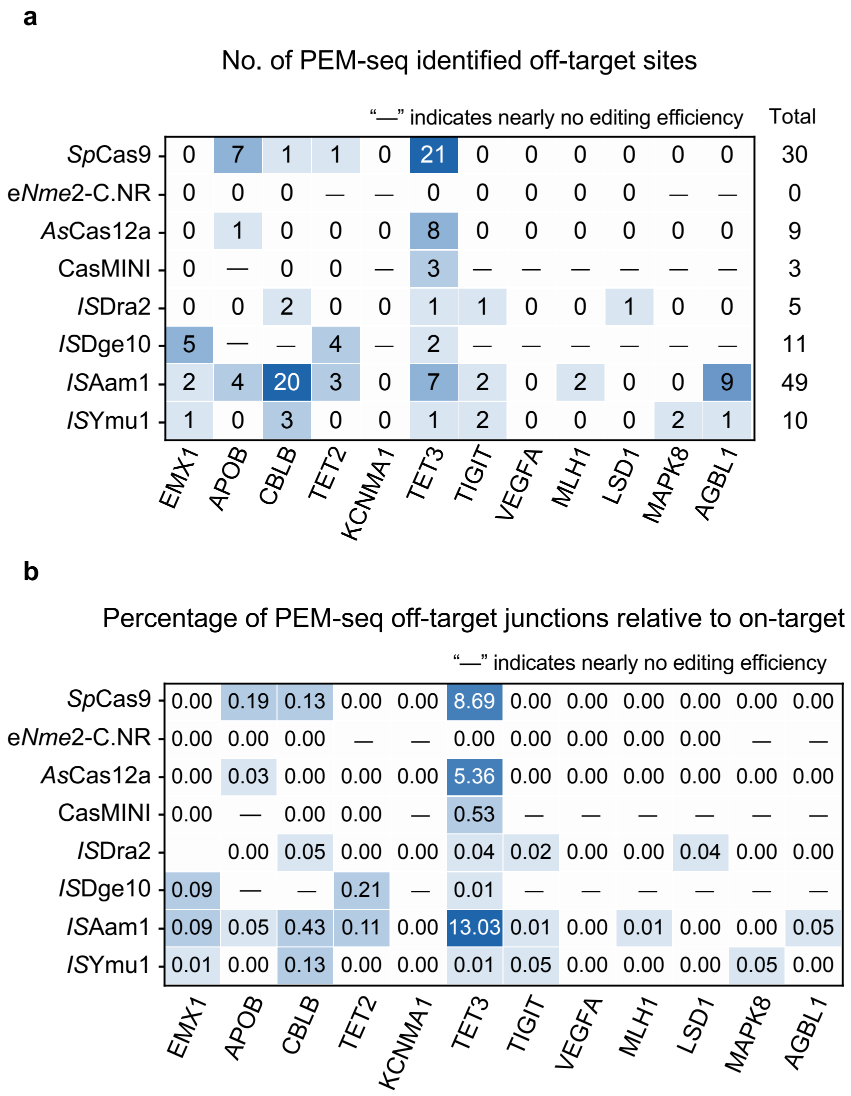

Figure S5. PEM-seq detected genome-wide editing off-targets of various nucleases. Related to Figure 4. (**a-b**) Summary heat map showing the detailed off-target site numbers (**a**) and the total off-target cleavage percentages (**b**) of eight representative TnpB/Cas12/Cas9 nucleases at the indicated twelve loci as identified by PEM-seq. The symbol "—" indicates cases where the identification of true off-targets is challenging due to nearly undetectable editing efficiencies at these target sites.

Off-targets sequencing reads | *TET3*

*Sp*Cas9

Target	Spacer Sequence	PAM	Counts	Coordinate		
ON	TATGGTAAGACATGCAGACC	TGG	167020	chr2	74,097,473	74,097,495
OT1	GATGGTAAGCCATGCAGACC	TGG	2374	chr3	14,386,902	14,386,925
OT2	TATGGTAAGACATGCAGACA	TGG	2378	chr17	12,411,784	12,411,807
OT3	TATGGTAAGACATGCAGACA	AGG	1934	chr22	22,952,974	22,952,997
OT4	TTTGGTAAGACATGCAGACG	TGG	1707	chr2	44,574,107	44,574,130
OT5	TATGGTAAGACATGCAGACA	TGG	1616	chr6	17,054,667	17,054,690
OT6	TATGGTAAGACATGCAGACA	TGG	1274	chr12	19,660,985	19,661,008
OT7	TATGGTAAGACATGCAGACA	GAG	1419	chr2	47,294,253	47,294,276
OT8	TATGGTAAGACATGCAGACT	TGG	533	chr8	109,853,657	109,853,680
OT9	TATGGTAGGACATGCAGACA	TGG	379	chr1	110,473,506	110,473,529
OT10	ATATGGTAAGAATGCAGACC	TGG	324	chr2	130,589,473	130,589,495
OT11	ATATGGTAAGAATGCAGACC	TGG	289	chr2	130,531,275	130,531,297
OT12	TATGGTAAGACACACAGACA	TGG	104	chr2	71,169,511	71,169,534
OT13	TATGGTAAAACATGCAGACA	AGG	81	chr13	98,104,373	98,104,396
OT14	TATGGTAGGACATGCAGACA	TGG	58	chr11	132,629,252	132,629,275
OT15	CATGGTAAGACATGCAGACG	GGA	9	chr1	237,712,015	237,712,038
OT16	TACGGTAAGACATGCAGACA	TGG	9	chr1	208,728,730	208,728,753
OT17	TATGGTAAGACATGCAGGCC	CAG	7	chr10	80,589,980	80,590,003
OT18	ATAGGTAAGACACACAGACC	AGG	5	chr16	9,905,819	9,905,842
OT19	TATGGTAAGACACACAGACC	CAG	4	chr8	23,688,053	23,688,076
OT20	TTTGGTAAGACATGCAGACA	CAG	3	chr8	27,481,147	27,481,170
OT21	TATGGTAAGACATGCAGACA	CAG	6	chr3	131,739,503	131,739,526

*As*Cas12a/CasMINI

Target	PAM	Spacer Sequence	Counts		Coordinate		
			*As*Cas12a	CasMINI			
ON	TTTA	ATCGGATATGGTAAGACATG	179993	41116	chr2	74,097,463	74,097,486
OT1	TTTA	ATCAGATATGGTAAGACACA	7111	16	chr2	71,169,520	71,169,544
OT2	TTTA	ATCGGATATGGTAAGACATG	1611	143	chr6	17,054,657	17,054,681
OT3	TTTA	ATCGGATATGGTAAGACACG	595	58	chr18	46,708,876	46,708,900
OT4	TTTA	ATCAGATATGGTAAGACACA	155	0	chr6	116,841,487	116,841,511
OT5	TTTA	ATTGGATATGGTAAGACACA	113	0	chr3	127,731,859	127,731,883
OT6	TTTA	ATCAGGTATGGTAAGACATG	35	0	chr2	37,683,731	37,683,755
OT7	TTTA	ATCAGGTTTGGTAAGACATG	11	0	chr2	44,574,097	44,574,121
OT8	CTTA	ATCAGATATGGTAAGACATA	13	0	chr1	114,321,214	114,321,238

*IS*Dra2/*IS*Ymu1

Target	PAM	Spacer Sequence	Counts		Coordinate		
			*IS*Dra2	*IS*Ymu1			
ON	TTGAT	TTTAATCGGATATGGTAAGA	28249	79180	chr2	74,097,458	74,097,482
OT1	TTTAT	TTTAATCAGATATGGTAAGA	10	5	chr2	71,169,524	71,169,549

*IS*Dge10

Target	PAM	Spacer Sequence	Counts	Coordinate		
ON	TTAT	CCAGGTCTGCATGTCTTACC	94911	chr2	74,097,476	74,097,499
OT1	TTAC	CCAGGTCTGCATGTAAGTTA	3	chr5	82,692,169	82,692,193
OT2	TTAT	CCAGGTCTCAACAGTTTGGT	3	chr2	27,929,238	27,929,262

*IS*Aam1

Target	PAM	Spacer Sequence	Counts	Coordinate		
ON	TTTAA	TCGGATATGGTAAGACATGC	24512	chr2	74,097,463	74,097,487
OT1	TTTAA	TCAGATATGGTAAGACACAC	2989	chr2	71,169,519	71,169,544
OT2	TTTAA	TCGGATATGGTAAGACATGC	96	chr6	17,054,657	17,054,682
OT3	TTTAA	TCGGATATGGTAAGACACGA	79	chr18	46,708,875	46,708,900
OT4	TTTAA	TCAGATATGGTAAGATATGC	13	chr4	15,685,174	15,685,199
OT5	TTTAA	TCAGATATGGTAAGATACCA	8	chr1	60,042,161	60,042,186
OT6	TTTAA	CCAGATATGGTAAGAATGCA	4	chr2	130,589,479	130,589,504
OT7	TTTAA	CCAGATATGGTAAGAATGCA	4	chr2	130,531,265	130,531,290

Figure S6. Sequence alignments and reads counts of PEM-seq detected genome-wide editing off-targets of various nucleases at *TET3* locus. Related to Figure 4.

Sequence alignments and reads counts of PEM-seq detected genome-wide off-targets at the *TET3* locus. The off-target sequences and relative reads counts, genomic coordinates are listed. Of note, the translocation junctions within 100 bp of the identified off-target were included in the corresponding off-target counts, and the mismatched bases are highlighted in red in the figure.

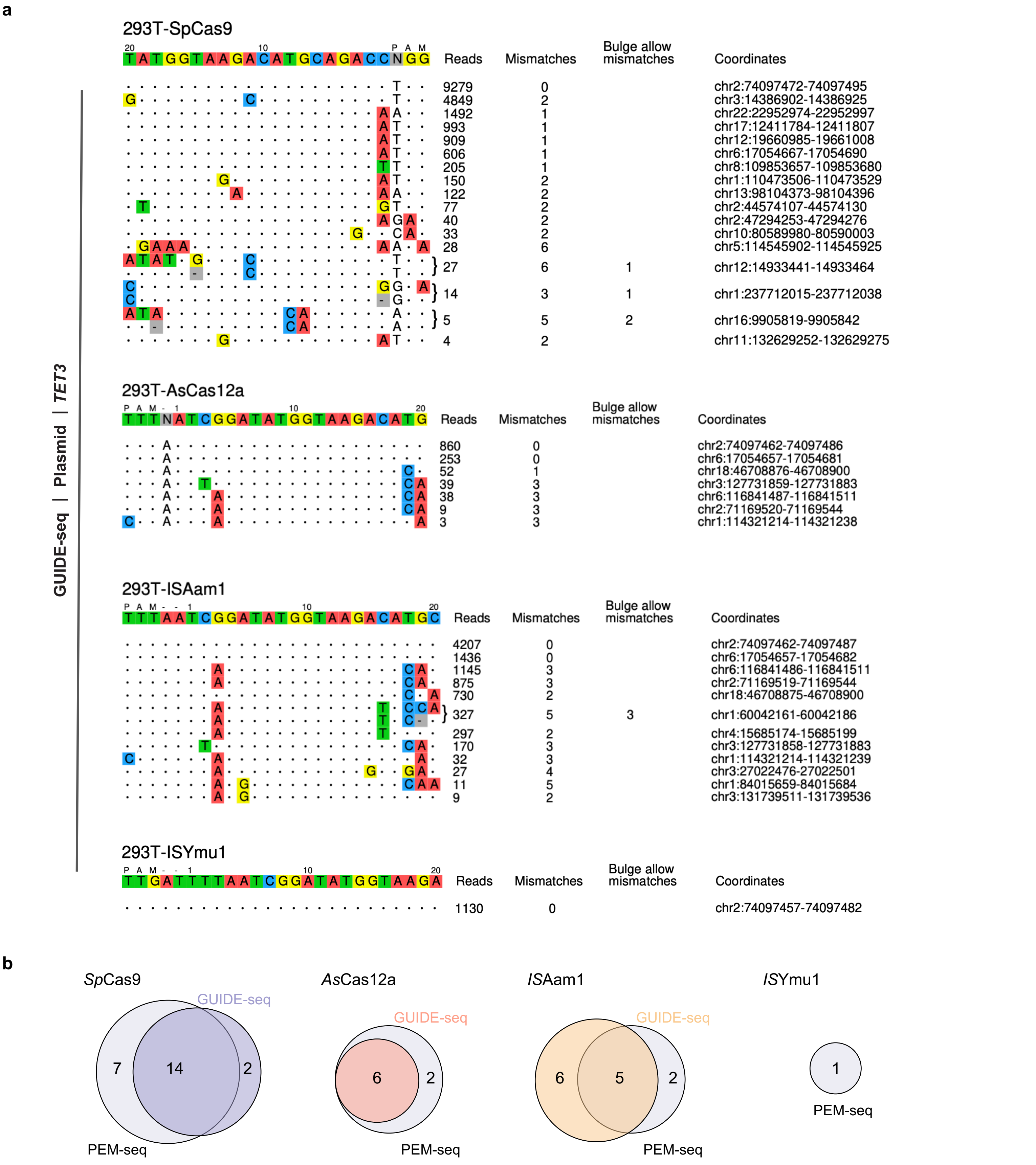

Figure S7. GUIDE-seq detected genome-wide editing off-targets of different nucleases. Related to Figure 4.
(**a**) Sequences of off-target sites at *TET3* locus following plasmid transfection identified by GUIDE-seq. The intended target sequence is presented at the top, with off-target cleaved sites below and mismatches with the on-target site highlighted in color. GUIDE-seq reads counts and genomic coordinates are shown on the right.
(**b**) Comparison of off-target site identification by PEM-seq and GUIDE-seq. Analysis was performed for *Sp*Cas9, *As*Cas12a, *IS*Aam1, and *IS*Ymu1 at the *TET3* locus. Venn diagrams illustrate the overlap of off-target sites detected by each method.
