# Supplementary material for: Comprehensive assessment of activity, specificity, and safety of hypercompact TnpB systems for gene editing
Source: Genome Biol. 2026 Jan 21;27:39. doi: 10.1186/s13059-026-03949-8 (PMC12908284; doi:10.1186/s13059-026-03949-8)
Supplement: Supplementary file 2 — Additional file 2: Table S1. Bio-primer, Red-primer, and gRNA sequences used in PEM-seq assay. Table S2. gRNA sequences and NGS primers design in Target-sequencing assay. Table S3. gRNA sequences and NGS primers design in Target-sequencing assay. [file 13059_2026_3949_MOESM2_ESM.pdf]

Table S1. Bio-primer, Red-primer, and gRNA sequences used in **PEM-seq** assay.

Site	Bio-primer		Nested-primer	
EMX1	GTAGACCTAGACTACAGACCGTCAC		CCTAGCTCTGAGCCATAGACC	
gRNA	*Sp*Cas9	AGAGTGGCCTTGATTTGTAC	*IS*Dra2/Ymu1	TTGTACAGGCATCACTTTAG
	Nme2-C.NR	AAGAGTGGCCTTGATTTGTACAG	*IS*Dge10	TTTAAAGAGTGGCCTTGATT
	Cas12a/f	AAGAGTGGCCTTGATTTGTA	*IS*Aam1	AGAGTGGCCTTGATTTGTAC
APOB	ACTGTTTGAATCCTGCTCTGC		CAGGAAAACTCACAGTGACC	
gRNA	*Sp*Cas9	CATGTATCAAGCACAGTGCT	*IS*Dra2/Ymu1	ACATGAATAGTAAGTGCCTG
	Nme2-C.NR	TTCATGTATCAAGCACAGTGCTG	*IS*Dge10	CAGGCACTTACTATTCATGT
	Cas12a/f	AAGCACCCAGCACTGTGCTT	*IS*Aam1	AGCACCCAGCACTGTGCTTG
CBLB	GCTCTAGCTTTGCCTCAGAGC		GTAAAATGAGATAACTGGGTATC	
gRNA	*Sp*Cas9	ATATCTTCCCAAGTTAAACC	*IS*Dra2/Ymu1	ATCTTCCCAAGTTAAACCAG
	Nme2-C.NR	TATCTTCCCAAGTTAAACCAGGA	*IS*Dge10	CCTGGTTTAACTTGGGAAGA
	Cas12a/f	ACTTGGGAAGATATCAATGA	*IS*Aam1	CTTGGGAAGATATCAATGAG
TET2	GGTATACATGAACCCTACCTTAC		GCCCAAATATCTTCATCTAAGG	
gRNA	*Sp*Cas9	ATCATGAATATAAACATCAA	*IS*Dra2/Ymu1	GTTTATATTCATGATATTAA
	Nme2-C.NR	TCATGATATTAATGTAATGTCTT	*IS*Dge10	ATTCATGATATTAATGTAAT
	Cas12a/f	AAGACATTACATTAATATCA	*IS*Aam1	AGACATTACATTAATATCAT
KCNMA1	GCAGCAGTCCCTGTAGCTTTCAG		GGGGCATTCTGGGAAAGGATG	
gRNA	*Sp*Cas9	CGCTGCTACTTCTTATGCTA	*IS*Dra2/Ymu1	TCATGTTTAAACGCTGCTAC
	Nme2-C.NR	GCTGCTACTTCTTATGCTACGGT	*IS*Dge10	GCTACGGTTACGCGGATTAT
	Cas12a/f	AACGCTGCTACTTCTTATGC	*IS*Aam1	ACGCTGCTACTTCTTATGCT
TET3	CTTTAATATGCAAAGATGACTTC		CTCGCAAAAGAAGGAAGACAG	
gRNA	*Sp*Cas9	TATGGTAAGACATGCAGACC	*IS*Dra2/Ymu1	TTTAATCGGATATGGTAAGA
	Nme2-C.NR	GTAAGACATGCAGACCTGGATAA	*IS*Dge10	CCAGGTCTGCATGTCTTACC
	Cas12a/f	ATCGGATATGGTAAGACATG	*IS*Aam1	TCGGATATGGTAAGACATGC
TIGIT	GGCCTATAGATGTTAGAAATGGGTC		CTCCTCTGAGAACCAGAGAAG	
gRNA	*Sp*Cas9	TAAACTAACCCCATCTTAAG	*IS*Dra2/Ymu1	TTAAACTAACCCCATCTTAA
	Nme2-C.NR	ACTAACCCCATCTTAAGTGGTTG	*IS*Dge10	-
	Cas12a/f	AACTAACCCCATCTTAAGTG	*IS*Aam1	ACTAACCCCATCTTAAGTGG
VEGFA	GAACAGTGGGGAAACAGGCCAGTCC		CCACGAAGGGTTACGGCAAAG	
gRNA	*Sp*Cas9	CATCTTCTCCCCTATCGACT	*IS*Dra2/Ymu1	TAGTCATCTTCTCCCCTATC
	Nme2-C.NR	TAGTCATCTTCTCCCCTATCGAC	*IS*Dge10	-
	Cas12a/f	AAGCCAAGTCGATAGGGGAG	*IS*Aam1	AGCCAAGTCGATAGGGGAGA
MLH1	CATAGGGAGAGAACCGGGTTCAGAG		GCTACTCAATAGGTGCCTCAG	
gRNA	*Sp*Cas9	AGTCTTTAAGCAAGTCTATG	*IS*Dra2/Ymu1	GAGAAGTCTTTAAGCAAGTC
	Nme2-C.NR	GAAGTCTTTAAGCAAGTCTATGG	*IS*Dge10	-
	Cas12a/f	AGCAAGTCTATGGGGTTCAA	*IS*Aam1	GCAAGTCTATGGGGTTCAAT
LSD1	CCCCTTTGAGATACATATTTGACC		GCTATACTTCGGATTTTCAGG	

gRNA	*Sp*Cas9	AAGATGTAGCTTCTAGCAAC	*IS*Dra2/Ymu1	GACTAAGGTAAGATGTAGCT
	Nme2-C.NR	TACATCTTACCTTAGTCATCAAC	*IS*Dge10	-
	Cas12a/f	ACCGGTTGCTAGAAGCTACA	*IS*Aam1	CCGGTTGCTAGAAGCTACAT
MAPK8	CCTCAGGTTCCTCAATGCTCTCTG		GACAATTTCCCAGACATCCTG	
gRNA	*Sp*Cas9	TTTCACTTGATGCTTTAGCT	*IS*Dra2/Ymu1	GCTTTAGCTAGGATAATTTA
	Nme2-C.NR	TCCTAGCTAAAGCATCAAGTGAA	*IS*Dge10	CCTAGCTAAAGCATCAAGTG
	Cas12a/f	AATTATCCTAGCTAAAGCAT	*IS*Aam1	ATTATCCTAGCTAAAGCATC
AGBL1	CCTACCCTGTCCTCCCAGACAGCTC		CTCCACCTTTCCCATTTCTAG	
gRNA	*Sp*Cas9	GCTTAAATTATTGATCCTCG	*IS*Dra2/Ymu1	GATTGTTCTGCTTAAATTAT
	Nme2-C.NR	GATGATTGTTCTGCTTAAATTAT	*IS*Dge10	-
	Cas12a/f	AGCAGAACAATCATCAACAT	*IS*Aam1	GCAGAACAATCATCAACATC

Table S2. gRNA sequences and NGS primers design in **Target-sequencing** assay (***Sp*Cas9 vs. *IS*Aam1**).

**Site**	**NGS-Out F' primer**	**NGS-Out R' primer**
TET3	GAGGGTTCAAAACCACAAGAG	GCAAATTCTTCCCTAGATGGAAC
gRNA	ACATCTGAAGGCCTACTGTT	
MLH1	CTTCTGTTCAGGTGGAGGACC	CAATGTATGAGCACTAGAACAC
gRNA	AAAATCCAAGTGAAGAATAT	
EMX1	GTTCTTATTGTTAGGAAGAAC	GTTAATATCTGTATCTGTCTCCAG
gRNA	TGAGAGCAGACAGAAGTATA	
AGBL1	GTTAAACTAGATTCTTCCTCTGC	GGTATAGAATTGACGCCTAGGC
gRNA	TTAATTTGTTTTGTAGAGGT	
APOB	GGCTTCTCAACGGCATCTCTCA	CAGCCAGGACTTGGATGCTTAC
gRNA	TTGGGGAGTCTAGTAGAGTT	
MAPK8	CCACCACCAATCCCACCATCC	CATCTGGAAACTTATTAGAGGTAC
gRNA	TGTGCATCAGAATCACATGG	
KCNMA1	CTTCAGCCATGACTCAGGAAC	CTTCTTTCCCCCAGTTTATTGC
gRNA	GTACACAGACACAAACACGG	
LSD1	CTCCAGTGAGTATCTCCGGG	CCTCCATGTTGCCTTCCTGTC
gRNA	CCCTTTAGGATTCCTTTGAA	

Table S3. gRNA sequences and NGS primers design in **Target-sequencing** assay (***Sp*Cas9 vs. *IS*Dra2/*IS*Ymu1**).

Site	NGS-Out F' primer	NGS-Out R' primer
MLH1	GCTTCAACAATTTACTCTCCC	GGGATTAGTATCTATCTCTCTAC
gRNA	TGCCAGCACATGGTTTAGGA	
EMX1	GGTTGGATCTGTGCAGCCAC	GACCACCTCCCCTGACCATC
gRNA	TATTCAAGTGGCGCAGATCT	
CBLB	GAATCTGAGCAATCAATAATTGTC	CTCCAGATCCCACATCTAAGC
gRNA	CTACCTTTGGTGAACCCGTT	
TET2	GAGGCTTATGTTGCAAAAGGTG	CCAAAAGCTCATGTGCAGTC
gRNA	TCAAGTGCTGTTTCAACACT	
APOB	GAGATAGGTGTGGACTGAGGG	GACTGCCTGTTCTCAATGAGAG
gRNA	TCATGACTTGGCAATAACTC	
DNMT3B	AACTCCTGGCCTCAAGTGAG	AACCTTGGAAAAGTAAATGAAGTAGAAAGCTTG
gRNA	GTTAGGATTAGGTGGGCTCA	
PGK1	TGGAACAAGTAGAATCTGTAGGCAAGAG	TTACAGGGAGGAATATCTTTGGACAGAG
gRNA	ACTCTCTCCTCAATGCCCTC	
MECP2	AGAGGGTGTGCAGGTGAAAAG	TCACGGCTTTCTTTTTGGCCTC
gRNA	CACCATGACCTGGGTGGATG	
